# Multifocal Langerhans cell histiocytosis in an adult with a pathological fracture of the mandible and spontaneous malunion: A case report

**DOI:** 10.3892/ol.2014.2272

**Published:** 2014-06-20

**Authors:** SAILANG SHI, YANMING LIU, TAO FU, XIUZHEN LI, SHIFANG ZHAO

**Affiliations:** 1 Dental Clinic, Sir Run Run Shaw Hospital, School of Medicine, Zhejiang University, Hangzhou, Zhejiang 310016, P.R. China; 2 Department of Oral and Maxillofacial Surgery, Second Affiliated Hospital, Zhejiang University School of Medicine, Hangzhou, Zhejiang 310009, P.R. China; 3 Department of Pathology, Second Affiliated Hospital, Zhejiang University School of Medicine, Hangzhou, Zhejiang 310009, P.R. China; 4 School of Dentistry, Zhejiang University, Hangzhou, Zhejiang 310006, P.R. China

**Keywords:** Langerhans cell histiocytosis, mandible, malunion, pathological fracture

## Abstract

Langerhans cell histiocytosis (LCH) is rare in the adult population and even rarer with jaw involvement. The current study presents the case of a 39-year-old male who complained of recurrent pain, swelling of the gingiva and an occasional pus-like discharge in the right mandible for one year. The patient was previously prescribed antibiotics, but this did not resolve the problem. An initial panoramic radiograph showed an osteolytic lesion and bone fracture in the right mandible. Eight months later, a new radiograph showed the spontaneous malunion of the fractured mandible. The patient was eventually diagnosed with Langerhans cell histiocytosis by histopathology and immunohistochemistry. Further lesions were found in the ribs and ilium by nuclear bone scanning. The patient was subsequently treated with systemic chemotherapy, and the lesions are currently effectively being controlled. This study is the first to show that spontaneous intralesional bone regeneration may lead to reunification of the mandible fracture caused by LCH in an adult.

## Introduction

Langerhans cell histiocytosis (LCH), formerly termed histiocytosis X, is a disease characterized by the neoplastic proliferation of Langerhans cells (1). LCH is rare in the adult population, and additional multifocal involvement is even rarer (2–5). LCH includes three subtypes: Eosinophilic granuloma (EG), Hand-Schuller-Christian disease and Letterer-Siwe disease. EG is the major type, accounting for 60–70% of all LCH cases; it is a localized form and presents as unifocal or multifocal bone lesions (6,7).

Adult LCH may involve the temporal bone and jaws (8,9). In extremely rare cases, the jaws involved may be fractured due to continuous enlargement of the osteolytic lesion. The present study reports a case of LCH in an adult male in which multiple bones (mandible, rib and pelvis) were involved. In this case, the mandible was pathologically fractured and spontaneously healed eight months later. To the best of our knowledge, no similar case has ever been reported. Therefore, the clinical, radiographical and histopathological features of this rare case are highlighted. Patient provided written informed consent.

## Case report

A 39-year-old male presented with a one-year history of pain, swelling of the gingiva and an occasional pus-like discharge in the right mandible. Several teeth had slowly become loose and one tooth had fallen out. The patient was previously prescribed antibiotics by a local dentist who considered the problem to be a bacterial infection. The symptoms were alleviated, yet the problem was never completely resolved. Eight months prior to the current presentation, an initial panoramic radiography of the jaw was taken in a local hospital and the patient was diagnosed with osteomyelitis of the jaw. Although it was suggested that the patient should receive further treatment at a tertiary hospital, since the symptoms were tolerable, this advice was not followed in the eight months previous to the current presentation. At this time, the patient was immediately admitted to the Department of Oral and Maxillofacial Surgery, Second Affiliated Hospital, Zhejiang University School of Medicine (Hangzhou, China) for further investigation.

**Figure 1:**
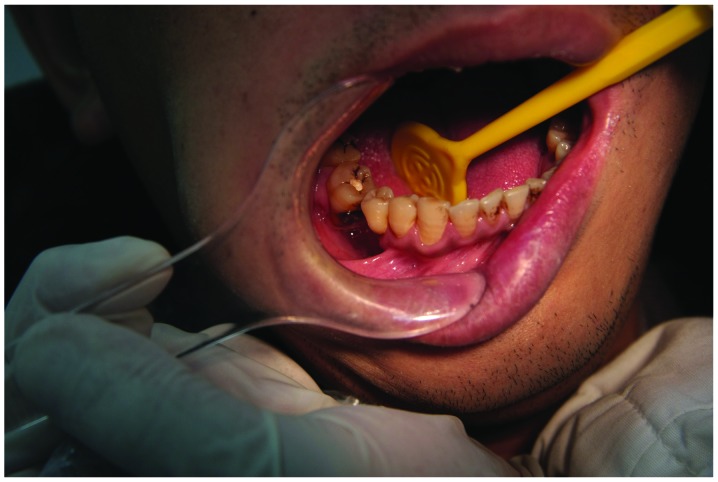
A pit in the right mandible. No pus is being discharged from the pit. The first molar is missing and the other molars are sloped anteriorly.

Clinical palpation of the right mandible revealed that the lateral surface of the mandibular body bulged and that the inferior margin of the body was concave. The first molar was missing and mobility of the neighboring teeth was detected. The second and third molars sloped anteriorly, resulting in immature tooth contact. There was a conspicuous pit in the right mandible, between the first premolar and the second molar, yet no obvious pus-like discharge was found (Fig. 1). The midline of the mandible was shifted to the right by ~2 mm.

**Figure 2:**
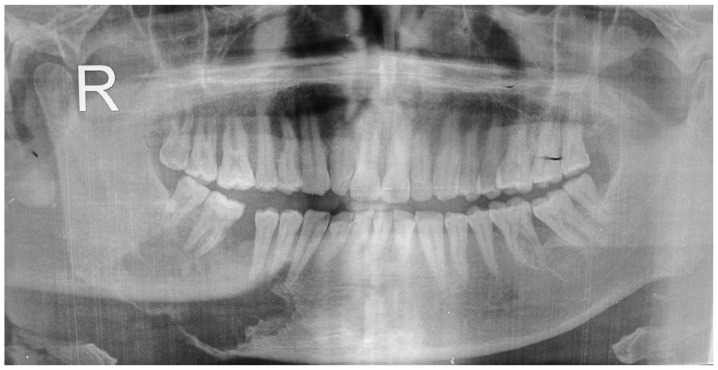
Initial panoramic radiograph showing a radiolucent lesion with a moth-eaten margin in the right mandible. The lesion resulted in the fracture of the mandible. The involved teeth appear to be floating on the lesion.

**Figure 3:**
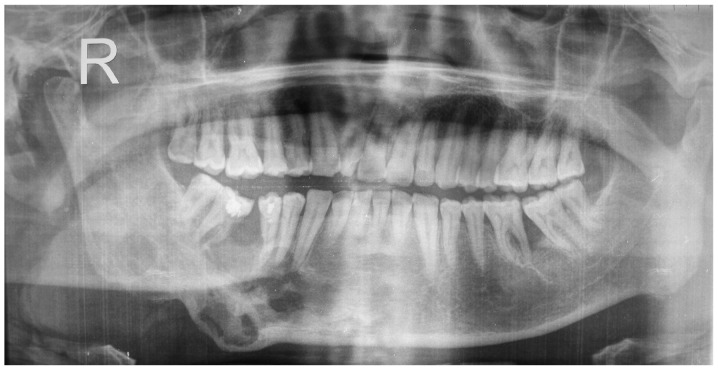
Second panoramic radiograph showing intralesional bone regeneration and malunion of the fractured mandible eight months later.

**Figure 4:**
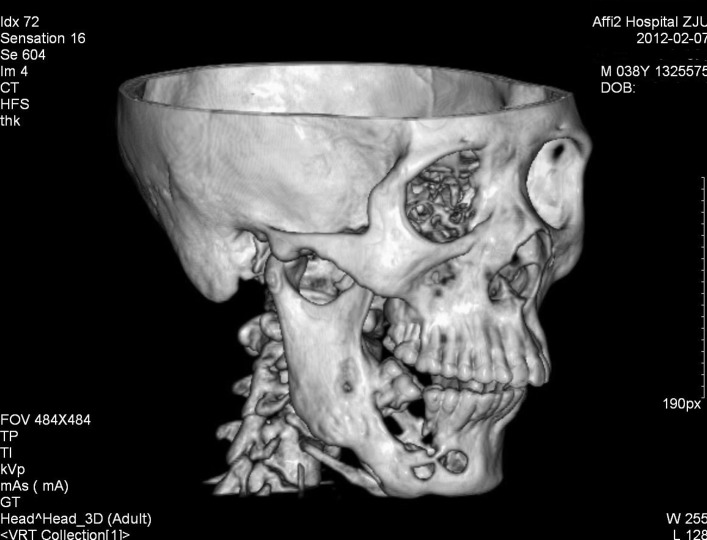
Computed tomography (CT) scan confirming the continuity of the mandible eight months later.

The initial panoramic radiograph showed an osteolytic lesion in the right mandible, ranging from the canine to the third molar, and with a moth-eaten margin. The lesion had already invaded the cortices and resulted in a pathological fracture of the mandible (Fig. 2). As the supporting bone was destroyed, the involved teeth appeared to be floating in the osteolytic lesion. The displacement of bone fragments led to immature contact of the lower third molar with the upper second molar (Fig. 2). A second panoramic radiograph taken at the Second Affiliated Hospital, Zhejiang University School of Medicine (Hangzhou, China) showed that eight months later, although the lesion had continued to expand slightly, there was apparent new bone regeneration in the previous osteolytic area, which had resulted in a malunion of the fractured bone segments (Fig. 3). The continuity of the right mandible was also confirmed by computed tomography (CT) scanning (Fig. 4).

**Figure 5:**
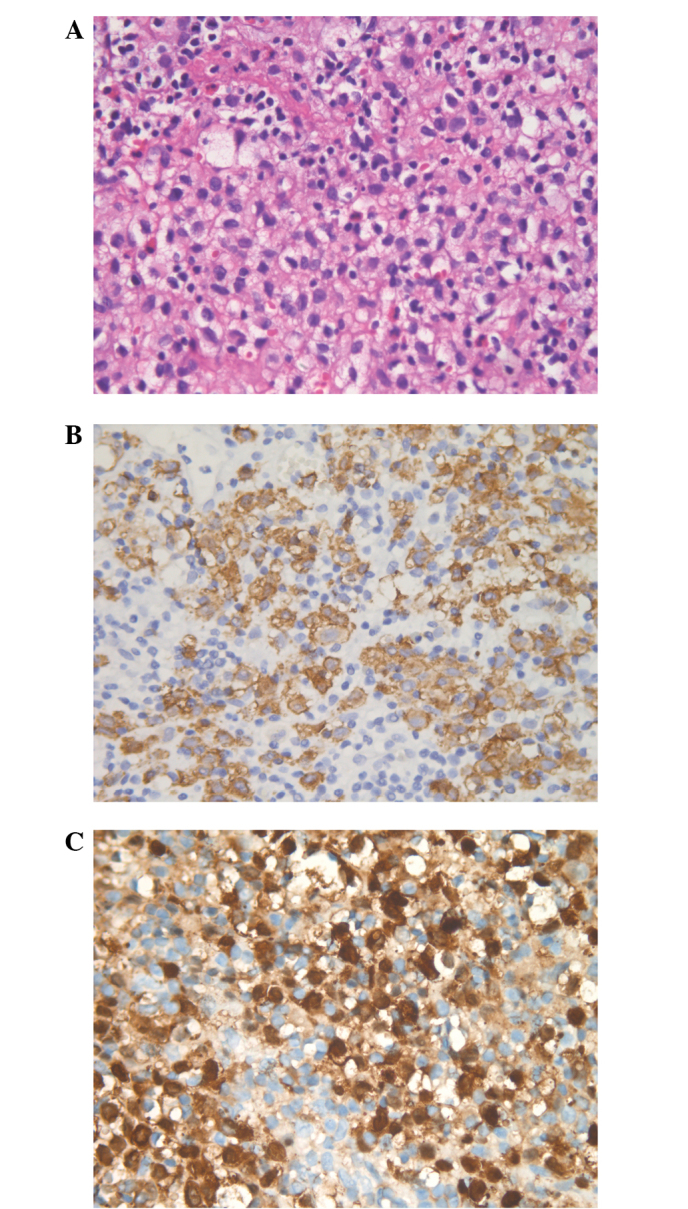
Clusters of Langerhans cells with (A) distinct folded or grooved nuclei, resembling a coffee bean. (B) Cells demonstrate positive immunoreactivity to cluster of differentiation (CD)1a. (C) Cells demonstrate a positive immunoreactivity to S-100 protein. Hematoxylin and eosin staining; original magnification, ×400.

To establish a diagnosis, an incisional biopsy was performed. This showed clusters of medium-sized cells with coffee bean-like nuclei that were folded or grooved; the characteristic feature of Langerhans cells. A few eosinophils were also found around these characteristic cells (Fig. 5A). These clusters of cells were finally identified as Langerhans cells by their intense immunoreactivity for S-100 protein and cluster of differentiation (CD)1a (Fig. 5B and C). The diagnosis of LCH was consequently confirmed.

**Figure 6:**
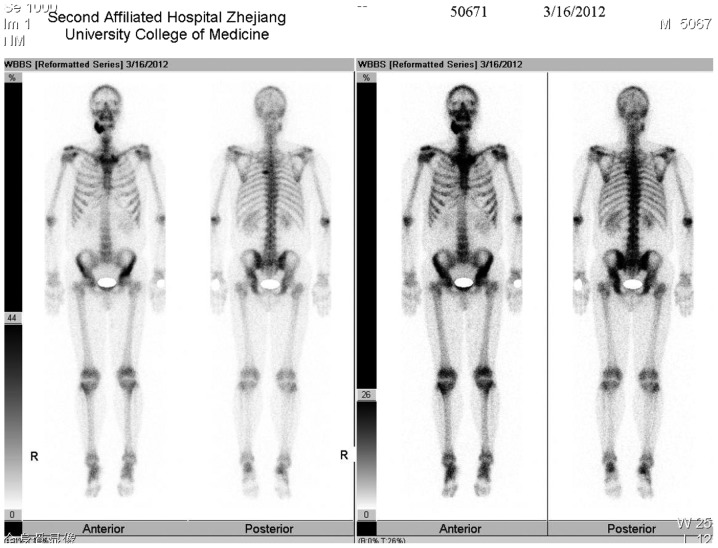
Nuclear bone scanning showing the increased uptake of technetium-99m in the right mandible, left fifth rib and left ilium, indicating multiple bones are involved in the LCH in this case.

**Figure 7:**
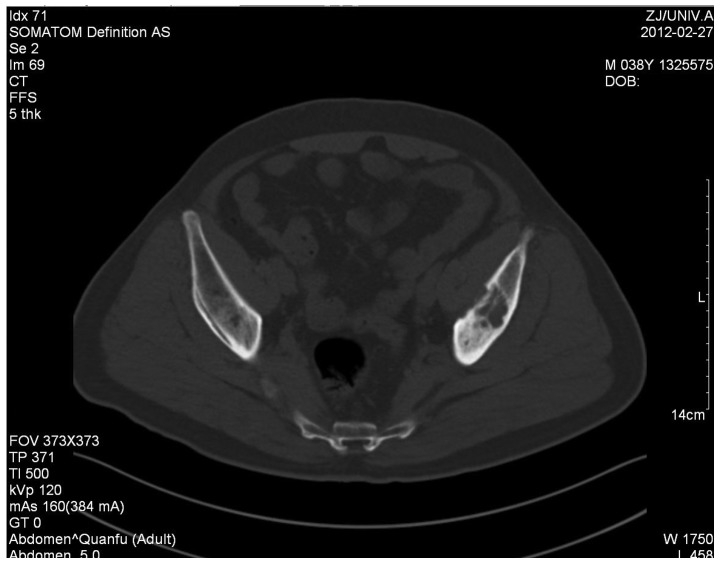
Computed tomgraphy (CT) scan of the pelvis demonstrating an osteolytic lesion in the left ilium.

Nuclear bone scanning with technetium-99m was then performed to investigate whether other bones were involved; besides the right mandible, the left ilium and the left fifth rib also showed increased uptake of the radionuclide (Fig. 6). CT scanning also confirmed the osteolytic focus in the left ilium (Fig. 7). No more organs were found to be involved by either chest radiography or magnetic resonance (MR) scanning of the abdomen. The patient was finally diagnosed with LCH with multifocal bone lesions.

As multiple bones were involved, surgical oblation or curettage was not the first treatment option. The patient was referred to the hematological department and received combination chemotherapy. The chemotherapy lasted for nine months and was divided into six courses. In each course the patient was administeres 750 mg etoposide, 160 mg vincaleukoblastine and 1.47 g prednisone.

## Discussion

In the present rare case, multiple bones were involved in LCH in an adult patient. It was noteworthy that the mandible involved was fractured due to the enlargement of the osteolytic lesion, and was re-united simultaneously, without any special medical interference, eight months later. This case presents valuable clinical evidence that intralesional bone regeneration may occur under natural conditions in an adult with LCH.

LCH is a rare disease, with an incidence of five cases per million individuals and one to two cases per million adults (1–5). Various organs may be involved, including the liver, skin, lungs, lymph nodes, bone marrow and spleen. However, the bone is the organ most frequently involved. The jaws are reported to be affected in 10–20% of all LCH cases (10,11) and the mandible is three times more frequently involved than the maxilla. The posterior mandibular region is the most frequently affected region (10).

The common clinical manifestations of LCH in the maxillofacial region include intraoral masses or swelling, pain, gingivitis, ulcers of the mucosa and loss of/loose teeth due to the erosion of the supporting bone tissue (10,12). Radiographically, these osteolytic jaw lesions tend to have a well-defined radiolucent appearance. Erosion of the bone around the teeth often gives the appearance that the teeth are floating on the radiolucent lesion (7,10,12). Root resorption of the involved teeth may occur, but is not common. A radiographical differentiation diagnosis is necessary between LCH and other osteolytic diseases, including osteomyelitis, osteosarcoma, odontogenic cysts and giant cell granuloma.

In the present case, a biopsy showed there were clusters of cells with a reniform nucleus that was deeply indented or grooved like a coffee bean. This is the typical feature of Langerhans cells, the hallmark cells of LCH. Immunohistochemesitry showed that these cells were positively stained for S-100 protein and CD1a antibodies. Generally, a biopsy is mandatory to differentiate LCH from other osteolytic malignant tumors. A presumptive diagnosis is made when the typical morphological features of Langerhans cells are found, and confirmed if stains for S-100 protein and CD1a antigen are both positive. In addition, the finding of Birbeck granules under electric microscope also confirms the diagnosis (5). Once a diagnosis of LCH has been established, it is necessary to perform further examinations, such as nuclear bone scanning, CT and MR imaging, to decide if occult lesions exist in other skeletal sites or other organs. This is an important aspect of the planning of a definitive treatment.

Treatment modalities for adult LCH include surgical curettage or resection, irradiation, local drug injection and systemic chemotherapy. These methods may be used either alone or in combination. With regard to a focal bone lesion, surgical curettage or bone resection remain choices for treatment (13,14). Local radiation may aid in the relief of symptoms, but seldom achieves complete remission when used alone (7). Intralesional drug injections may be an effective option for a localized LCH. Libicher *et al* reported cases of complete remission of a solitary bone lesion within 6 months following a single local application of methylprednisolone (15). Other studies reported satisfactory results with intralesional steroid injections or indomethacin (16–19). With regard to a soft tissue lesion or multifocal bone lesions however, systemic chemotherapy is often the first choice (20). A previous study has even recommended multiagent chemotherapy for bone-only LCH, whether unifocal or multifocal, based on clinical data that single agent chemotherapy or irradiation is associated with a higher recurrence rate (10). The prognosis of LCH is related to the age of patients at the time of onset. Generally, the prognosis is poorer in young patients and improved in elderly patients. The prognosis is also associated with the number of organs involved, being poorer when multiple organs are affected (21).

The current study presented a case of LCH with multiple bone lesions in an adult. Considering the chief complaints, it could easily have been misdiagnosed as an inflammatory disease. The radiographical appearance of an osteolytic lesion with a moth-eaten margin in the mandible was similar to that of an osteosarcoma. This case highlighted the fact that LCH should be one of the differential diagnoses for an osteolytic lesion of the jaw in an adult. The panoramic radiograph showed that the mandible was fractured and displaced due to enlargement of the lesion. However, a second radiograph showed that the fracture had been mal-united eight months later and that a quantity of new bone had formed in the previous radiolucent cavity. This indicated that the patient had a relatively good prognosis. However, since there were other lesions in the rib and ilium, multiagent chemotherapy was necessary. The effectiveness of the treatment was proven by the findings of recent CT scans. However, resection of the mandibular lesion and immediate reconstruction with autogenous bone grafting can be reserved for later use, in case chemotherapy fails to result in complete healing. Also, an osteotomy of the mandible may be performed later to correct the malocclusion.

In summary, the present study documented the clinical and radiographical manifestations of an unusual case of LCH in an adult. In this case, multiple bones were involved and a pathological mandibular fracture occurred. For the first time, this case provides evidence that spontaneous intralesional bone regeneration exists and may subsequently lead to reunification of the fractured mandible in an adult patient with LCH.

## References

[1] Nicholson HS, Egeler RM, Nesbit ME (1998). The epidemiology of Langerhans cell histiocytosis. Hematol Oncol Clin North Am.

[2] Favara BE, Feller AC, Pauli M (1997). Contemporary classification of histiocytic disorders. The WHO Committee On Histiocytic/Reticulum Cell Proliferations Reclassification Working Group of the Histiocyte Society. Med Pediatr Oncol.

[3] Aricò M, Girschikofsky M, Généreau T (2003). Langerhans cell histiocytosis in adults. Report from the International Registry of the Histiocyte Society. Eur J Cancer.

[4] Coppes-Zantinga A, Egeler RM (2002). The Langerhans cell histiocytosis X files revealed. Br J Haematol.

[5] Leonidas JC, Guelfguat M, Valderrama E (2003). Langerhans’ cell histiocytosis. Lancet.

[6] Piattelli A, Paolantonio M (1995). Eosinophilic granuloma of the mandible involving the periodontal tissues. A case report. J Periodontol.

[7] dos Anjos Pontual ML, da Silveira MM, de Assis Silva Lima F, Filho FW (2007). Eosinophilic granuloma in the jaws. Oral Surg Oral Med Oral Pathol Oral Radiol Endod.

[8] Alexander RL, Worthen ML, Pang CS, May JS (2013). Langerhans cell histiocytosis: temporal bone invasion in an adult. Ear Nose Throat J.

[9] Yepes JF, Khalaf M, Cunningham L (2012). Chronic focal Langerhans cell histiocytosis of the left mandibular condyle presenting as limited jaw opening: a case report. Ear Nose Throat J.

[10] Hicks J, Flaitz CM (2005). Langerhans cell histiocytosis: current insights in a molecular age with emphasis on clinical oral and maxillofacial pathology practice. Oral Surg Oral Med Oral Pathol Oral Radiol Endod.

[11] Neville BW, Damm DD, Allen CM (2002). Hematologic disorders. Oral and Maxillofacial Pathology.

[12] Eckardt A, Schultze A (2003). Maxillofacial manifestations of Langerhans cell histiocytosis: a clinical and therapeutic analysis of 10 patients. Oral Oncol.

[13] Alexiou GA, Mpairamidis E, Sfakianos G, Prodromou N (2009). Cranial unifocal Langerhans cell histiocytosis in children. J Pediatr Surg.

[14] Bartnick A, Friedrich RE, Roeser K, Schmelzle R (2002). Oral Langerhans cell histiocytosis. J Craniomaxillofac Surg.

[15] Libicher M, Roeren T, Tröger J (1995). Localized Langerhans cell histiocytosis of bone: treatment and follow-up in children. Pediatr Radiol.

[16] Esen A, Dolanmaz D, Kalayci A (2010). Treatment of localized Langerhans’ cell histiocytosis of the mandible with intralesional steroid injection: report of a case. Oral Surg Oral Med Oral Pathol Oral Radiol Endod.

[17] Moralis A, Kunkel M, Kleinsasser N (2008). Intralesional corticosteroid therapy for mandibular Langerhans cell histiocytosis preserving the intralesional tooth germ. Oral Maxillofac Surg.

[18] Park JW, Chung JW (2010). Long-term treatment of Langerhans cell histiocytosis of the mandibular condyle with indomethacin. Oral Surg Oral Med Oral Pathol Oral Radiol Endod.

[19] Putters TF, de Visscher JG, van Veen A, Spijkervet FK (2005). Intralesional infiltration of corticosteroids in the treatment of localised langerhans’ cell histiocytosis of the mandible: Report of known cases and three new cases. Int J Oral Maxillofac Surg.

[20] Liu YH, Fan XH, Fang K (2007). Langerhans’ cell histiocytosis with multisystem involvement in an adult. Clin Exp Dermatol.

[21] Muramatsu T, Hall GL, Hashimoto S (2010). Clinico-pathologic conference: case 4. Langerhans cell histiocytosis (LCH). Head Neck Pathol.

